# Bioinspired Multilayer Silicone Composites: Autonomous Healing and Rate-Dependent Mechanics via Dynamic Boron Coordination Networks

**DOI:** 10.3390/polym17223040

**Published:** 2025-11-17

**Authors:** Hongwen Zeng, Yan Peng, Tao Liu, Lijuan Zhao, Fengshun Zhang

**Affiliations:** 1 College of Chemistry and Materials Science, Sichuan Normal University, Chengdu 610066, China; 20231201054@stu.sicnu.edu.cn; 2 Institute of Chemical Materials, China Academy of Engineering Physics, Mianyang 621010, China; yanpengcaep@163.com (Y.P.); liutaocaep@163.com (T.L.)

**Keywords:** diboron, siloxane, multilayer structure, self-healing, rate response

## Abstract

Inspired by the cutaneous wound healing mechanism observed in human scab formation, we engineered a series of multilayered silicone rubber composites through alternating polydimethylsiloxane (PDMS) and polydiborosiloxane (PDBS) laminates. The dynamic diboron–oxygen coordination bonds within PDBS enabled both autonomous self-healing through bond reconfiguration and enhanced impact resistance via energy dissipation. PDMS served dual functions as both a structural reinforcement matrix and a flow-restricting framework for PDBS, thereby improving the viscoelastic creep behavior and irreversible deformation tendencies characteristic of conventional non-Newtonian fluids. Notably, increasing the laminate count from 3 to 9 layers enhanced structural integration, yielding improvement in dimensional stability. All multilayer configurations demonstrated remarkable healing performance, achieving post-24 h self-healing efficiencies exceeding 95% across 3-layer, 5-layer, and 9-layer specimens. Rheological characterization revealed pronounced strain rate sensitivity under multiaxial loading conditions, with storage modulus showing proportional enhancement to applied strain rates in both transverse and longitudinal orientations.

## 1. Introduction

Silicone rubber is widely used in electrical and electronic devices, the aerospace industry, and healthcare due to its excellent thermal stability, resistance to cold and chemical corrosion, as well as its superior electrical insulation properties [1,2]. However, in practical applications, silicone rubber materials inevitably undergo mechanical stress and material aging, which can lead to a decline in performance or even structural damage [3,4]. Conventional silicone rubber materials often struggle to recover their original properties after damage, thereby limiting their service life and application scope [5].

Self-healing mechanisms can be categorized into two primary types: external and intrinsic self-healing. External self-repairing involves incorporating functional substances (such as microcapsules or microvascular networks) onto the surface or inside the material. When damage occurs, the repair agents are released to facilitate the repair process. However, the extent and efficacy of healing are contingent upon the specific external substances employed [6,7,8]. In contrast, intrinsic self-healing exploits a material’s inherent reversible chemical bonds or dynamic interactions. These bonds can undergo repeated cleavage and reformation, enabling autonomous repair [9]. Representative interactions and chemical bonds include imine bonds [10,11], Diels–Alder (DA) adducts [12,13], disulfide bonds [14], ester bonds [15,16,17], hydrogen bonds [18,19,20], coordination bonds [21,22], siloxane equilibrium [23,24], and ionic interactions [25,26], amongst others. Self-healing mechanisms can also be categorized into two distinct classes based on their operational modes: autonomous and stimuli-responsive. Autonomous self-healing refers to the capability of a material to spontaneously repair damage, such as cracks, without external energy input. This process is typically initiated merely by direct contact between the fractured interfaces. In contrast, stimuli-responsive self-healing requires the application of external stimuli—such as heat, light, electricity, or specific chemical environments—to activate the internal repair mechanism. In the absence of such stimuli, the self-healing process cannot proceed. Current research in self-healing elastomer design has evolved beyond these singular mechanisms. Increasingly, designs incorporate two or more mechanisms to synergistically enhance healing efficiency and mechanical performance. Cui et al. introduced a novel self-healing and recyclable rubber composite structure, utilizing imine bonds and Cu^2+^–imine crosslinking within a polybutadiene rubber matrix, complemented by Cu^2+^–imidazole crosslinking at the silica–rubber interface. The highly dynamic nature of the imine bonds and Cu^2+^-N (Cu^2+^–imidazole and Cu^2+^–imine) complexes imparted exceptional self-healing and recyclability to the material, achieving a self-healing efficiency of up to 90% (in terms of tensile strength) and high recycling rates [27]. Yan et al. developed a self-healing polydimethylsiloxane (PDMS) elastomer by incorporating dual-reversible bonds (imine bonds and hydrogen bonds) into the siloxane chains. The resulting PDMS films exhibited remarkable stretchability (>4000%), superior transparency (>80%), and rapid self-healing efficiency, with a recovery rate of 93% at room temperature [28].

Polyborosiloxane (PBS) demonstrates exceptional impact resistance and self-healing capabilities due to its unique reversible boron/oxygen coordination bonds [29]. At low strain rates, PBS exhibits a compliant, viscoelastic behavior characterized by reversible deformation. As the strain rate augments, the elastic modulus of PBS undergoes a steep increase, leading to material stiffening and consequently conferring superior impact resistance [30,31]. The intrinsic self-healing capacity of PBS is primarily attributed to the dynamic boron/oxygen coordination bonds embedded within its structure. During the self-healing process, the molecular chains of PBS undergo rearrangement, facilitating the recombination of fractured boron/oxygen coordination bonds. This rebonding mechanism not only mitigates surface damage but also rejuvenates the internal structural integrity of the material [32,33].

PBS containing dynamic boronic ester bonds exhibits distinct differences in both structural features and dynamic behavior compared to systems based on boroxine (B_3_O_3_) rings. In PBS networks, boron primarily forms interchain crosslinks through “single-point” or “dual-point” bridges, resulting in a flexible, low-functionality three-dimensional architecture. The dynamic behavior in PBS mainly originates from boronic ester exchange reactions: existing B–O bonds undergo nucleophilic attack by water molecules, leading to a “break-then-reform” process via transesterification or hydrolysis–re-esterification pathways. This process has a relatively low activation energy and can occur even at room temperature [29,30,31,32,33]. In contrast, boroxine-based systems are constructed from B_3_O_3_ six-membered rings formed by the self-condensation of three boronic acid molecules. Each ring acts as a “one-ring–three-arm” high-functionality crosslinking node, imparting high mechanical strength to the material. The dynamic characteristics of such systems arise from the thermally reversible “ring-opening and re-closing” behavior of the B–O bonds within the boroxine rings. Upon heating, ring strain is released, leading to cleavage of the B–O bonds and the formation of intermediate species. Upon cooling, these intermediates can undergo dehydration again to re-form the boroxine rings, thereby establishing a “high-strength, high-stimulus-responsiveness” dynamic equilibrium [34,35]. Liang et al. successfully synthesized a self-healing silicone elastomer by leveraging a synergistic kinetic mechanism based on meticulously designed intermolecular and intramolecular nitrogen-coordinated boroxines. The resulting material exhibits robust mechanical properties and a highly autonomous self-healing capability, achieving a repair efficiency of approximately 96% after 48 h at room temperature [36]. Wu et al. synthesized a novel material through the dehydration condensation reaction between hydroxyl-terminated PDMS and diboronic acid. The incorporation of dynamic diboron–oxygen coordination bonds within the resultant polydiborosiloxane (PDBS) endowed the material with exceptional impact resistance and self-healing properties at room temperature [37].

Compared to traditional PBS, the diboron structure introduced in PDBS results in stronger diboron–oxygen coordination bonds, thereby conferring superior performance in properties such as impact resistance [37]. The self-repair process in most self-healing materials requires direct contact between the fractured interfaces or an external stimulus to initiate the subsequent healing mechanisms. To address this limitation, we have formulated a model inspired by the natural healing process of skin scabbing by devising a multilayered structural configuration. In this work, we have synthesized PDBS with diboron structures using hydroxyl-terminated silicone oil and diboronic acid, and subsequently constructed a multilayered alternating architecture via a controlled layering strategy. In this design, PDMS replicates the combined roles of muscle tissue and blood clots, leveraging its covalently crosslinked network architecture to provide structural reinforcement while establishing preliminary stabilization during the initial phase. Conversely, PDBS, possessing dynamic properties akin to flowing blood, performs the critical repair function. To delve into the intricacies of structural influence, various layer configurations have been meticulously designed, allowing for an in-depth exploration of how differing structural arrangements modulate the overall performance of the material.

## 2. Materials and Methods

### 2.1. Materials

Hydroxyl-terminated silicone oil, possessing a viscosity of 42 Pa·s, was procured from Zhonghao Chenguang Chemical Research Institute Co., Ltd., Zigong, China. Analytical-grade diboronic acid was procured from Adamas Reagent Co., Ltd., Shanghai, China. PDMS, exhibiting a molecular weight within the range of 630,000–750,000 g/mol and containing a vinyl content of 0.13–0.2%, was procured from Zhejiang Xinan Chemical Industrial Group Co., Ltd., Zhejiang, China. Silica (T36-5) was procured from Tonghua Shuanglong Chemical Co., Ltd., Jilin, China. Analytical-grade 2,5-dimethyl-2,5-(tert-butylperoxy)hexane was procured from Chengdu Kelong Chemical Co., Ltd., Chengdu, China. Analytical-grade methanol was procured from Chengdu Kelong Chemical Co., Ltd., Chengdu, China. Analytical-grade ethyl acetate was likewise purchased from Chengdu Kelong Chemical Co., Ltd., Chengdu, China.

### 2.2. Preparation Process of PDBS

**Figure 1:**
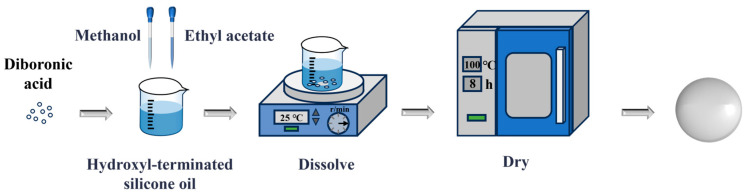
Schematic representation of the preparation process of PDBS.

The preparation procedure for PDBS is depicted in Figure 1. First, hydroxyl-terminated silicone oil and diboric acid were mixed in a specific molar ratio of 100:6 and subsequently dissolved in a blend of methanol and ethyl acetate at ambient temperature. The resulting solution was stirred for 3 h at room temperature to ensure homogeneous mixing. Subsequently, the mixture underwent vacuum drying at a temperature of 100 °C for 8 h. This step was crucial for the removal of solvent residues and the promotion of the formation of the polymer network, ultimately yielding the desired PDBS.

### 2.3. Preparation Process of Multilayer Silicone Rubber

**Figure 2:**
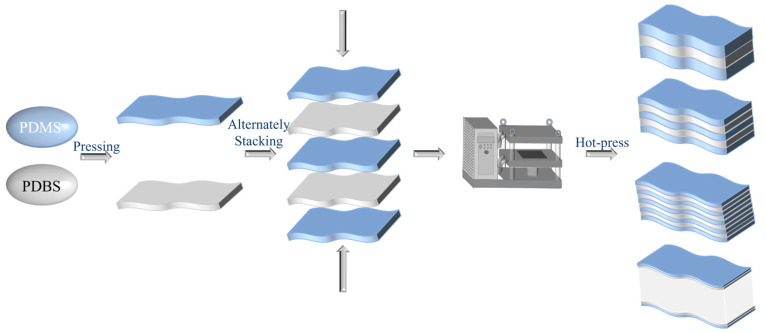
Preparation process of multilayer silicone rubber.

Figure 2 elucidates the comprehensive preparation methodology for the multilayer self-healing silicone rubber material. Initially, 100 g of PDMS, accompanied by 25 g of fumed silica and 1 g of a crosslinking agent (2,5-dimethyl-2,5-(tert-butylperoxy)hexane), were meticulously mixed and compounded utilizing a high-precision double-roll rubber mill (Collin Roll Mill W110, COLLIN Lab & Pilot Solutions GmbH, Maitenbeth, Germany) at a rotational speed of 5 r/min. Multiple passes were conducted to guarantee an even and homogeneous mixture. Subsequently, PDMS and PDBS were individually processed in the double-roll mill, where they were reduced to the desired thickness under ambient temperature conditions at a reduced speed of 1 r/min. The resultant rubber compounds were then sequentially layered in an alternating fashion to create a composite structure, with the number of layers being variable. These composite structures were subsequently cut into rectangular specimens of dimensions 40 mm × 120 mm × 2 mm and positioned within molds for the curing process. The curing operation was conducted in a platen vulcanizer (Laboratory Platen Press P300E, COLLIN Lab & Pilot Solutions GmbH, Maitenbeth, Germany) at an elevated temperature of 180 °C for 10 min, ultimately yielding a multilayer silicone rubber material with an alternating layered structure.

### 2.4. Characterization

Fourier transform infrared (FTIR) spectroscopy analysis was conducted utilizing the spectrometer (TENSOR 27 FT-IR spectrometer, Bruker Optik GmbH, Ettlingen, Germany), configured in attenuated total reflectance (ATR) mode, across a spectral range spanning from 4000 cm^−1^ to 500 cm^−1^. The subjects of this analysis included diboric acid, hydroxyl-terminated silicone oil and PDBS.

Scanning electron microscopy (SEM) analysis was conducted using the microscope (SH-5000M Scanning Electron Microscope, HIROX Co., Ltd., Tokyo, Japan) to examine the microstructure of the samples. Prior to examination, thin cross-sectional slices were obtained from the prepared alternating multilayer silicone rubber samples via a blade. Subsequently, the fractured surfaces were sputter-coated with gold in a vacuum chamber. Characterization of the samples ensued under an accelerating voltage of 15 kV, with a magnification of 40×.

Optical microscopy analysis was performed using the microscope system (KH-8700 Microscope System, HIROX, Co., Ltd., Tokyo, Japan) to observe the structural morphology of the alternating multilayer silicone rubber material. Thin cross-sections of the prepared samples were obtained using a blade and subsequently examined under an optical microscope at a magnification of 50×. Additionally, the structural changes within the material were monitored following tensile and drop-weight impact tests.

A cyclic tensile test was conducted utilizing the universal testing machine (TSE503A Universal Testing Machine, Shenzhen Wance Testing Equipment Co., Ltd., Shenzhen, China), with a stretching speed of 200 mm/min. The samples, shaped in a dumbbell configuration with dimensions of 12 mm × 2 mm × 35 mm, were prepared accordingly. The tests were executed at various time intervals, namely 30 min, 60 min, 2 h, 3 h, 12 h, and 24 h. A detailed analysis of the test results and self-healing efficiency ensued. The self-healing efficiency (*η*) is calculated by the following formula:

(1) $$\eta \;\left(\%\right)=\frac{S_{1}}{S_{0}}\times 100\%$$

where *S*_1_ and *S*_0_ are the hysteresis areas of healed and original, respectively.

A tensile test was conducted using a universal testing machine (TSE503A Universal Testing Machine, Shenzhen Wance Testing Equipment Co., Ltd., Shenzhen, China), with stretching speeds including 1 mm/min, 10 mm/min, and 100 mm/min. The samples, in a dumbbell shape with dimensions of 12 mm × 2 mm × 35 mm, were prepared for testing. To mitigate random errors, each sample group underwent five replicate tests, and the resultant data were subjected to statistical analysis. The material was molded into circular specimens, with a diameter of 5 mm and a thickness of 2 mm.

A compression test was executed using the solid-state rheometer (RSA3 Solid Rheometer, TA Instruments, New Castle, DE, USA) under ambient conditions, with strain rates including 0.01 mm/min, 0.1 mm/min, and 1 mm/min. Furthermore, cyclic compression tests were conducted at a strain rate of 0.1 mm/min, with the results analyzed accordingly.

The stress relaxation test employed the solid-state rheometer (RSA3 Solid Rheometer, TA Instruments, New Castle, DE, USA) to apply a strain of 10% and a normal force of 0.5 N at room temperature. The operation was conducted within the linear viscoelastic region of the sample. The stress relaxation behavior was recorded over a duration of 5 min. The relaxation modulus G_t_ was normalized by the initial relaxation modulus G_0_ and plotted against time. Following the Maxwell model framework, the characteristic relaxation time τ(T) was determined as the time at which G_t_/G_0_ decays to 1/e.

The drop-ball impact test was employed to evaluate the energy absorption and cushioning capabilities of the samples, utilizing the tester (LC-716 Ball Impact Tester, Dongguan Licen Instrument Co., Ltd., Dongguan, China). The tests were executed at varying drop heights, with multiple samples undergoing impact tests. Comparisons of the impact responses among different samples at the same drop height were conducted to assess the cushioning performance and impact resistance properties of the material. For the stress relaxation test, circular specimens, with a diameter of 25 mm and a thickness of 2 mm, were prepared.

## 3. Results and Discussion

### 3.1. Synthesis and Characterization of Samples

**Figure 3:**
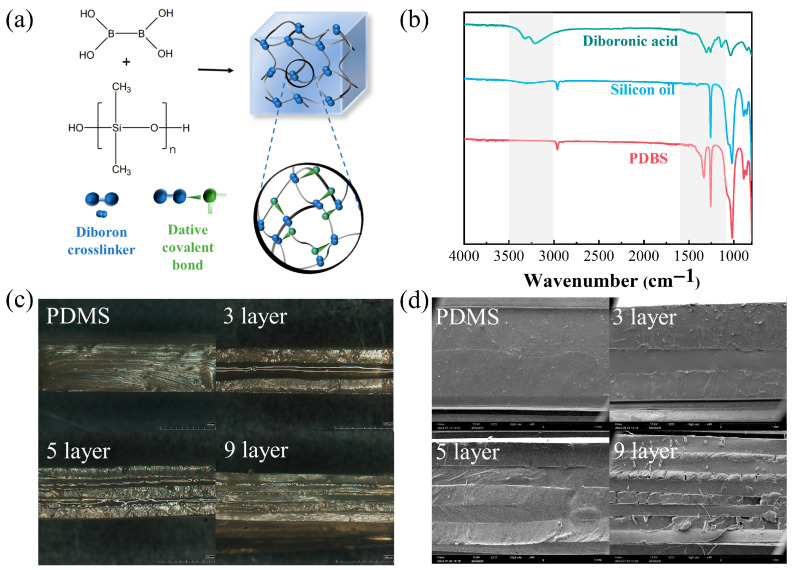
(**a**) Schematic representation of the preparation process of PDBS; (**b**) FTIR spectrum of PDBS; (**c**) optical microscopic characterization; (**d**) scanning electron microscopic (SEM) characterization of the multilayer structure.

The dehydration reaction between diboric acid and hydroxyl-terminated silicone oil, which produces PDBS containing diboron–oxygen coordinate bonds, is schematically depicted in Figure 3a. Comparative infrared spectroscopic analysis of the starting materials and the product was conducted to confirm the reaction, the results of which are presented in Figure 3b. In the FTIR spectrum, the characteristic doublet of B–OH (at approximately 3320 and 3220 cm^−1^) and the absorption peak of Si–OH (near 3300 cm^−1^) exhibited a noticeable decrease in intensity. Concurrently, the B–O stretching vibration shifted to a lower wavenumber from its original position at 1340 cm^−1^, while the antisymmetric Si–O–Si stretching peak at 1025 cm^−1^ became broadened and split. A new absorption emerged at 1020 cm^−1^, which can be attributed to the stretching vibration of the Si–O–B bond [38]. These spectroscopic changes collectively confirm the successful incorporation of boronic acid units into the siloxane backbone via covalent bonding, leading to the formation of a dynamic Si–O–B crosslinked network, and thus verifying the successful synthesis of the PDBS material. Alternate hot-pressing was then employed to fabricate the PDMS/PDBS laminates with varying numbers of layers, following the procedure illustrated in Figure 2. To elucidate the multilayer structure of the silicone rubber material, optical microscopy and scanning electron microscopy (SEM) were employed to inspect the cross-sections of the silicone rubber samples. The observational outcomes were illustrated in Figure 3c,d, which sequentially exhibited the cross-sections of pure PDMS and multilayer alternating structures comprising 3-layer, 5-layer, and 9-layer, respectively. With the overall thickness of the material remaining constant at 2 cm, the thickness of each layer in the multi-layer structure gradually decreases as the number of layers increases. Nevertheless, the layered structure maintained uniformity and integrity, with no notable damage or delamination discernible between the layers. Additionally, the layers demonstrated satisfactory interlayer bonding.

### 3.2. Self-Repairing Behavior and Mechanism Diagram

**Figure 4:**
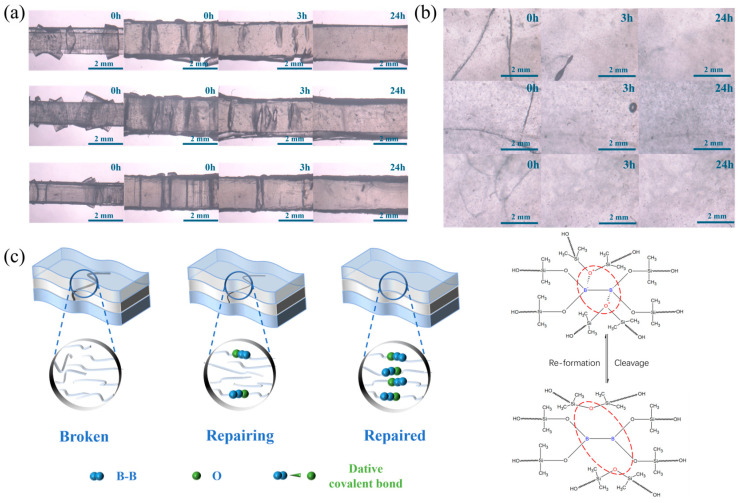
Optical microscopy characterization of multilayer self-healing: (**a**) stretching-induced healing process for 3-layer, 5-layer, and 9-layer structures; (**b**) drop-weight impact healing process for 3-layer, 5-layer, and 9-layer structures; (**c**) schematic illustration of the self-healing process.

The self-healing characteristics of the material were assessed through optical microscopic analysis. The pristine PDMS layer exhibited an absence of self-healing capability, whereas the PDBS layer demonstrated notable self-healing attributes. The multilayer structures, encompassing 3-layer, 5-layer, and 9-layer, underwent stretching and drop-weight impact test in both parallel and perpendicular orientations, as depicted in Figure 4a,b. During the stretching procedure, it was noted that the PDBS layers incurred fractures, but upon exposure to room temperature, the cracks progressively diminished over time. Specifically, after 3 h, a significant reduction in cracks was observed, and by 24 h, nearly complete healing of the cracks had occurred. The drop-weight impact healing mechanism exhibited a similar trend, with cracks becoming finer and healing occurring more rapidly as the layer count increased. As exemplified in Figure 4c, the PDBS layers within the multilayer configuration sustained fracture cracks. However, over time, these cracks underwent complete healing, thereby underscoring the exceptional self-healing proficiency of the material. These cracks form because, under high strain rates, the diboron–oxygen coordination bonds lack sufficient time to dissociate. This leads to localized stress concentration that exceeds the material’s fracture strength. This self-healing behavior is primarily attributed to the dynamic diboron–oxygen coordination bonds within PDBS, which possess the capacity to reform following fracture and dissociation, thereby enabling the material to autonomously repair itself whenever cracks or fractures arise at room temperature.

**Figure 5:**
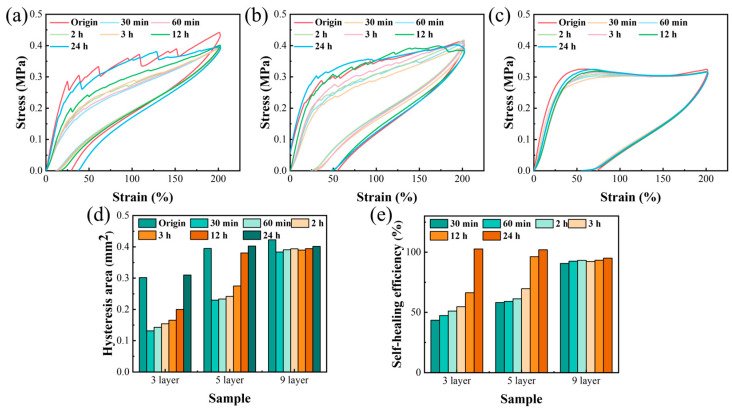
Cyclic tensile curves of multilayer self-healing materials: (**a**) 3-layer; (**b**) 5-layer; (**c**) 9-layer structures; (**d**) hysteresis loop area of the multilayer structure during cyclic tensile test; (**e**) self-healing efficiency of the multilayer structure.

To examine the specific effects of the alternating multilayer structure on the self-healing performance of the material, cyclic tensile tests were conducted using a universal mechanical testing machine. Throughout the cyclic tensile tests conducted using a universal mechanical testing machine, specimens were subjected to a specific deformation of 200% at a strain rate of 200 mm/min. The stress–strain curves obtained exhibited a serrated fluctuation pattern, which was indicative of crack formation within the PDBS layers. Nevertheless, with an increase in the number of layers, the structure became more uniform and intact, resulting in a decrease in the variability of the curves, as shown in Figure 5a–c. Furthermore, as demonstrated in Figure 5d,e, with an increase in the number of layers, the area of the hysteresis loop expanded, indicating an enhancement in energy dissipation. Moreover, with an increase in the healing duration of the PDBS layers, the area of the restored hysteresis loop also increased, indicating an augmentation in self-healing efficiency. The self-healing efficiency of the 3-layer, 5-layer, and 9-layer materials, respectively, attained 102%, 102%, and 95% after 24 h. It was noteworthy that the 9-layer configuration maintained reproducible cyclic tensile responses, with a consistent self-healing efficiency of over 90%.

### 3.3. Tensile Rate Response Characteristics

**Figure 6:**
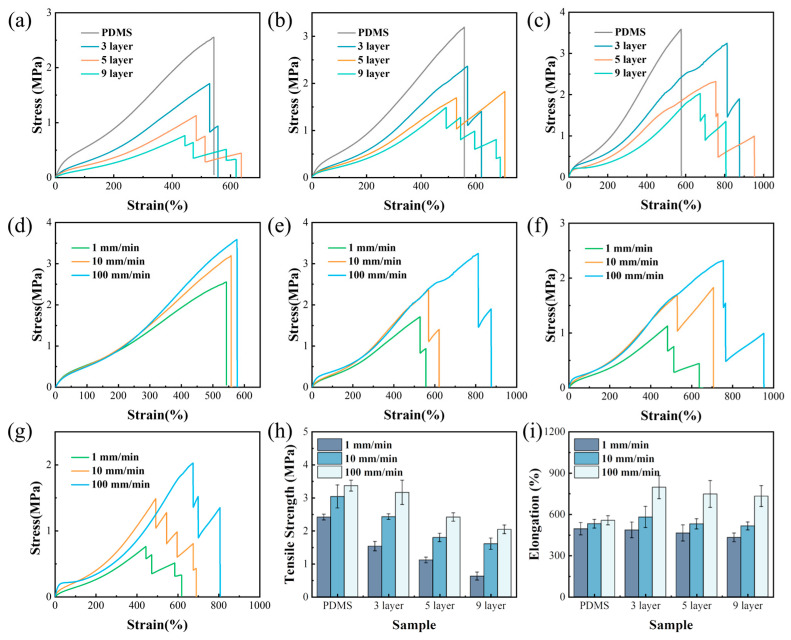
Tensile behavior of multilayer structures at different strain rates: (**a**) 1 mm/min; (**b**) 10 mm/min; (**c**) 100 mm/min. Stress–strain curves at different strain rates: (**d**) pure PDMS; (**e**) 3-layer; (**f**) 5-layer; (**g**) 9-layer; (**h**) fracture elongation of multilayer structures at different strain rates; (**i**) fracture strength of multilayer structures at different strain rates.

To elucidate the distinct lateral mechanical characteristics of multilayer silicone rubber materials, uniaxial tensile tests were executed at varying strain rates (1 mm/min, 10 mm/min, and 100 mm/min) utilizing a universal testing machine. The experimental findings, presented in Figure 6a–c, disclose a phenomenon of incremental delamination and fracture within the multilayer materials. Specifically, the monolayer of pure PDMS exhibited a single fracture event, whereas the 3-layer, 5-layer, and 9-layer structures underwent two, three, and five fracture incidents, respectively. Analysis of the stress–strain curve for the 3-layer structure revealed a notable stress reduction upon the fracture of the PDMS layer. However, subsequent to the fracture, as the PDMS layer retained its load-bearing capacity, the stress increased further with an increase in strain. Comparable delamination and fracture phenomena were also discernible in the other multilayer configurations. Notably, as the layer count escalated, the material exhibited a propensity towards softening, accompanied by a decrement in fracture strength.

Upon focusing specifically on the initial fracture event, the tensile tests performed across varying strain rates (as depicted in Figure 6d–i) demonstrate that, for samples possessing an equivalent layer count, the tensile fracture strength augmented with an increase in the strain rate. This augmentation could be attributed to the relaxation process inherent in tensile deformation. Specifically, at elevated strain rates, the molecular chains of the material were afforded insufficient time for relaxation, culminating in an enhanced fracture strength. Notably, this increase in fracture strength was more accentuated in multilayer materials due to the incorporation of PDBS layers. At lower strain rates, PDBS exhibited behavior akin to a viscous liquid, characterized by a low modulus and heightened flexibility. Conversely, at higher strain rates, it underwent a transition from a quasi-liquid state to a quasi-solid state, dissipating substantial energy and consequently augmenting the fracture strength of the material.

For samples with an equivalent number of layers, the tensile fracture elongation exhibited an ascending trend with an increase in strain rate. This phenomenon can be elucidated by bond dissociation kinetics, which posits that the fracture threshold of elastomers is dependent on the rate of deformation [39,40]. Under external tensile stress, chain tension diminishes the bond lifetime. Since bond dissociation constitutes an activation process necessitating thermal energy to surpass the dissociation barrier, elastomers within highly heterogeneous networks can undergo greater extension at elevated strain rates. At high strain rates, the temporal window available is markedly shorter than the lifetime of covalent bonds within the main chains, necessitating additional stretching to further abbreviate network lifetime until substantial bond dissociation ensues. Conversely, at low strain rates, the network lifetime surpasses the experimental timescale at comparatively minimal strain, leading to diminished elongation. Notably, the enhancement in elongation is even more accentuated in multilayer structures due to the incorporation of PDBS interlayers. At low strain rates, PDBS exhibits behavior akin to a viscous liquid, characterized by a low modulus and high compliance. However, under high strain rates, it undergoes a transition from a quasi-liquid to a quasi-solid state, dissipating considerable energy. Consequently, the increase in elongation observed in multilayer materials at higher strain rates is more pronounced compared to that observed in pure PDMS layers.

In the multilayer structure, the tensile load-bearing capacity predominantly relied on the pure PDMS layers. As the thickness of each PDMS layer progressively diminished within the structure, the tensile elongation and tensile strength exhibited a tendency to decrease with an increasing number of layers. As the number of layers increases, the thickness of the PDMS layers gradually decreases, and the force transfer in the layered structure becomes less effective. This leads to faster fracture during the tensile test and a gradual decrease in elongation. For identical samples tested under varying strain rates, the tensile fracture strength augmented as the strain rate escalated. The duration elapses at higher strain rates are significantly shorter compared to the lifespan of the covalent bonds in the main chains of the load-bearing structure. Consequently, additional stretching is needed to further abbreviate the network lifespan until a sufficient degree of bond dissociation is achieved. As a result, the tensile strength progressively intensifies with higher strain rates.

### 3.4. Compression Rate Response Characteristic

**Figure 7:**
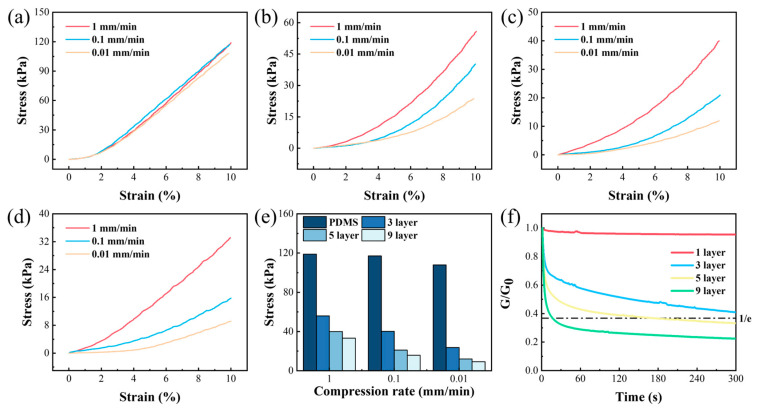
Compression behavior of multilayer structures at 20% strain under different strain rates: (**a**) PDMS; (**b**) 3-layer; (**c**) 5-layer; (**d**) 9-layer; (**e**) compression strength of different structures at various strain rates; (**f**) stress relaxation of different structures at 10% strain.

To elucidate the mechanical properties of the material in the vertical direction, compression tests were conducted utilizing a rotational rheometer at varied strain rates (0.01 mm/min, 0.1 mm/min, and 1 mm/min). The experimental data, depicted in Figure 7a–d, demonstrate that, at a compression rate of 0.1 mm/min and a compression strain of 10%, the multilayer structures exhibited an augmentation in compression strength as the compression rate escalated, whereas the monolithic PDMS layer displayed a consistent compression strength of approximately 110 N, exhibiting no such trend (as illustrated in Figure 7e). Under a compression deformation of 20%, the same rule also held true (as illustrated in Figure S8). As the layer count increased, the compression strength gradually diminished, which aligns with the results obtained from the tensile test. Figure 7f illustrates the stress relaxation behavior of structures with varying layer counts. The monolithic PDMS layer exhibited minimal to negligible relaxation, whereas the relaxation rate and extent augmented with an increasing number of layers, suggesting that the multilayer materials possess superior energy dissipation capabilities. This is because energy dissipation in the material is primarily facilitated by dynamic bonds in the PDBS layers. As the number of layers increased, the effective contact area between the PDBS and PDMS layers increased, resulting in faster force transfer and a tendency for increased energy dissipation. The light microscope images after compression relaxation are shown in Figure S10. As the number of material layers increased, the structure remained more intact.

### 3.5. Cyclic Compression Characteristics

**Figure 8:**
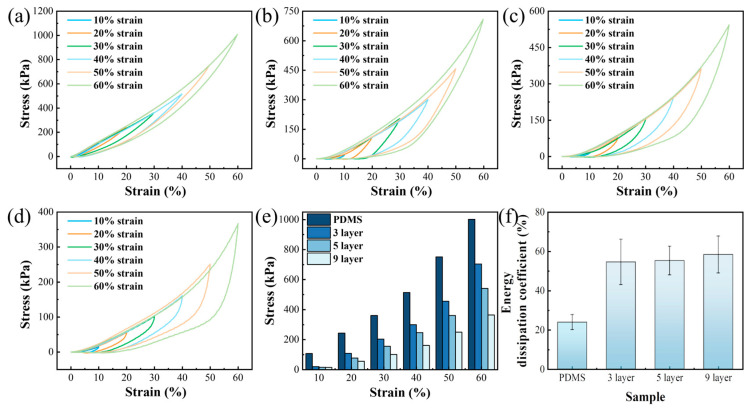
Compression curves at a rate of 0.1 mm/min: (**a**) PDBS; (**b**) 3-layer; (**c**) 5-layer; (**d**) 9-layer; (**e**) compression strength at different compressive strains for structures with varying layer numbers; (**f**) energy loss coefficients of structures with different layer numbers.

To assess the energy dissipation capacity of the material, cyclic compression tests were conducted on specimens with varying layer counts at different strain amplitudes, with a constant strain rate of 0.1 mm/min. The experimental outcomes, depicted in Figure 8a–f, reveal the influence of these variables. Considering the viscoelastic creep behavior of PDBS, minor deformation was inevitable during the tests. Consequently, distinct sections of the identical sample were subjected to repeated measurements to ensure accuracy. Notably, the resultant test curves exhibited substantial overlap, attesting to the excellent uniformity and reproducibility of the material. For a specified strain amplitude, an inverse relationship was observed between the compression strength during cyclic compression and the number of layers. The energy loss coefficient can be calculated by taking the ratio of the hysteresis area to the area under the loading curve. The detailed calculation figures are shown in Figures S1–S5. Specifically, the energy loss factor for the monolithic PDMS layer was relatively modest, at 0.24, whereas the loss factors for the 3-layer, 5-layer, and 9-layer structures increased to 0.54, 0.55, and 0.58, respectively. These findings underscored the superior energy dissipation capabilities of multilayer structures.

### 3.6. Impact Resistance Performance and Mechanism Diagram

**Figure 9:**
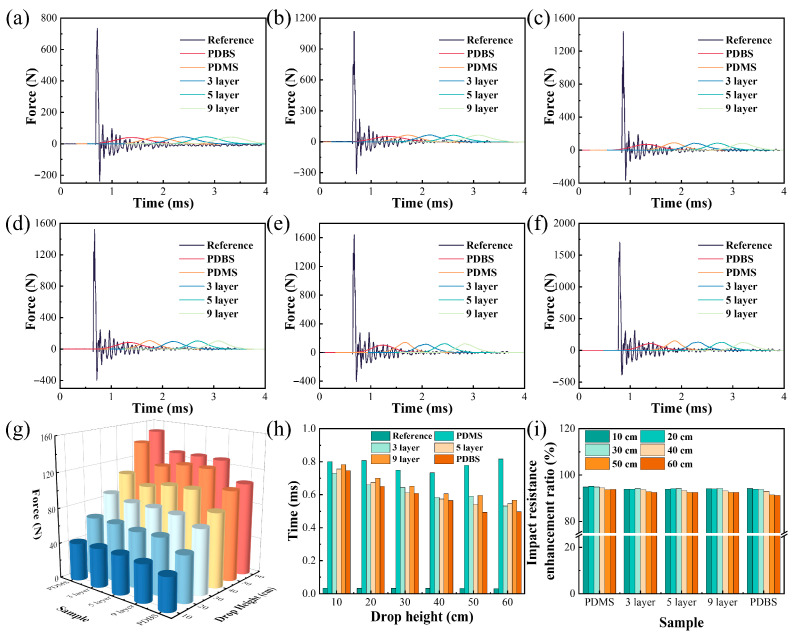
Force-time curves of different multilayer structures at varying drop heights: (**a**) 10 cm; (**b**) 20 cm; (**c**) 30 cm; (**d**) 40 cm; (**e**) 50 cm; (**f**) 60 cm; (**g**) peak impact force of different samples at varying drop heights; (**h**) buffering time of different samples at varying drop heights; (**i**) attenuation rate of different samples at varying drop heights.

To delve deeper into the influence of multilayer structures on the impact resilience of silicone rubber materials, drop-weight impact tests were performed on silicone rubber foam specimens. The assessment of impact resilience encompassed the analysis of the attenuation of the initial peak force and the variations in buffering duration, with the experimental outcomes presented in Figure 9a–i. Pure PDMS demonstrated notable impact resilience; however, the material exhibited inherent brittleness and catastrophic failure under impact loading. Consequently, the incorporation of additional PDMS layers effectively stabilized its morphology, thereby augmenting the overall structural stability of the material. As the drop height increased from 10 cm to 60 cm, the peak impact force of the reference group escalated from 732 N to 1705 N, accompanied by a buffering duration of 0.33 ms. Notably, at a drop height of 60 cm, the peak impact force of the 3-layer, 5-layer, and 9-layer structures decreased to approximately 127 N, marking a substantial 92% reduction in peak force in comparison to the reference group. The impact resistance enhancement ratio was defined as the difference between the reference impact force and the sample’s impact force, normalized by division by the reference impact force. Concurrently, the buffering duration increased to 0.53 ms, 0.54 ms, and 0.56 ms, respectively. During the rapid impact of the multilayer silicone rubber, the shear hardening effect of the PDMS layers appreciably bolstered the impact resilience.

**Figure 10:**
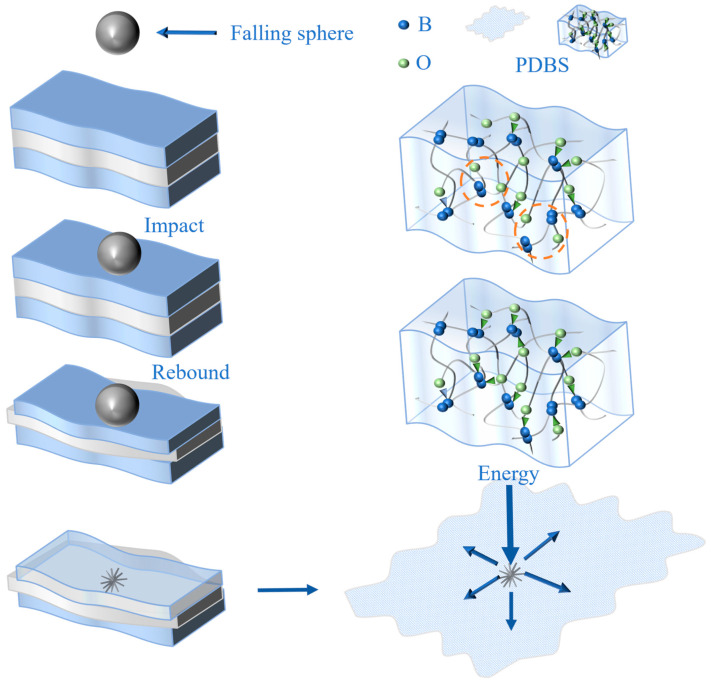
Schematic diagram of the impact resistance mechanism of multilayer structures.

As shown in Figure S9, after the ball impact test, the typical radial crack morphology was formed within the PDBS layer of the material. The dissipation mechanism of the impact resistance behavior is divided into two parts (as illustrated in Figure 10): Firstly, the PDMS matrix dissipates part of the impact energy through viscoelastic deformation. Then, the stress wave propagates to the lower layer of PDBS, causing local stress concentration and breaking through its fracture threshold. Moreover, the PDMS layer at the lower part will continue to dissipate part of the impact energy. Secondly, due to the fact that the diboron–oxygen coordination bonds in PDBS do not have sufficient time to relax, the total effective crosslink density remains unchanged over a short period. On the other hand, when they are forced to break, they can dissipate more energy. The alternating multilayer architecture serves as an effective buffer in force transmission, retarding the propagation of the impact force and, consequently, enhancing the protective efficacy. The buffering duration and impact energy dissipation rate of the multilayer structure reside within the range of those of the constituent phases, indicating the beneficial impact of the multilayer strategy. The fixed PDMS layer in the multilayer structure effectively improves the loss of effectiveness of the PDBS layer after fragmentation, and the addition of the PDBS layer also enhances the impact resistance of the pure PDMS layer. The same applies to the materials in the other layers.

## 4. Conclusions

In summary, we have developed a silicone rubber material possessing autonomous self-healing capabilities through a meticulously engineered multilayer structural design. Within the scope of this research, a novel PDBS featuring diboron structures was synthesized via a condensation reaction between hydroxyl-terminated silicone oil and diboronic acid. A PDMS/PDBS alternating multilayer architecture was subsequently constructed via a layer-stacking hot-pressing technique. The PDMS component significantly mitigated the viscoelastic creep behavior of PDBS, enabling the creation of a self-healing multilayer silicone rubber system. Optical microscopic analysis confirmed the excellent self-healing performance of the elastomer, while the cyclic tensile test revealed that the multilayer samples achieved self-healing efficiencies exceeding 90% within 24 h. Both tensile and compressive tests exhibited pronounced rate-responsive behavior: fracture strength, elongation at break, and compressive strength increased proportionally with strain rate. Furthermore, an increase in the number of layers was observed to accelerate stress relaxation, augment relaxation magnitude, and elevate energy dissipation, as evidenced by a higher loss factor. Drop-weight impact tests corroborated that the multilayer design thereby addressed the inherent brittleness of PDBS, significantly improving the impact resistance of the material. The dynamic diboron–oxygen coordination bonds in PDBS synergistically conferred the multilayer silicone rubber with robust self-healing capacity and exceptional impact tolerance. Ultimately, this work proposes a novel design strategy for self-healing materials that retains the inherent viscoelastic properties while effectively addressing the viscoelastic creep behavior limitations of conventional systems.

## Data Availability

The original contributions presented in this study are included in the article/Supplementary Materials. Further inquiries can be directed to the corresponding authors.

## Supplementary Materials

The following supporting information can be downloaded at: https://www.mdpi.com/article/10.3390/polym17223040/s1. Figure S1. Compressive modulus of structures with different numbers of layers under various cyclic compression conditions: (a) 10%; (b) 20%; (c) 30%; (d) 40%; (e) 50%; (f) 60%; Figure S2. Energy dissipation in a PDMS structure under different cyclic compression conditions: (a) 10%; (b) 20%; (c) 30%; (d) 40%; (e) 50%; (f) 60%; Figure S3. Energy dissipation in a 3-layer structure under different cyclic compression conditions: (a) 10%; (b) 20%; (c) 30%; (d) 40%; (e) 50%; (f) 60%; Figure S4. Energy dissipation in a 5-layer structure under different cyclic compression conditions: (a) 10%; (b) 20%; (c) 30%; (d) 40%; (e) 50%; (f) 60%; Figure S5. Energy dissipation in a 9-layer structure under different cyclic compression conditions: (a) 10%; (b) 20%; (c) 30%; (d) 40%; (e) 50%; (f) 60%; Figure S6. Compressive modulus of structures with different numbers of layers under various cyclic compression conditions: (a) PDMS; (b) 3-layer; (c) 5-layer; (d) 9-layer; Figure S7. Compressive modulus of different multi-layer structures under varying compressive strain: (a) 10%; (b) 20%; (c) 30%; (d) 40%; (e) 50%; (f) 60%; Figure S8. Compression behavior of multilayer structures at 20% strain under different strain rates: (a) PDMS; (b) 3-layer; (c) 5-layer; (d) 9-layer structures; (e) compression strength of different structures at various strain rates; Figure S9. Images of impact-damaged specimens from drop-weight tests with different layer configurations: (a) 3-layer; (b) 5-layer; (c) 9-layer; Figure S10. Optical microscopy images of compressive stress relaxation: (a) PDMS; (b) 3-layer; (c) 5-layer; (d) 9-layer.
