# Supplementary material for: Bioinspired Multilayer Silicone Composites: Autonomous Healing and Rate-Dependent Mechanics via Dynamic Boron Coordination Networks
Source: Polymers (Basel). 2025 Nov 17;17(22):3040. doi: 10.3390/polym17223040 (PMC12656262; doi:10.3390/polym17223040)
Supplement: Supplementary file 1 [file polymers-17-03040-s001.zip › polymers-3910565-supplementary.pdf]

Supporting Information

Bioinspired Multilayer Silicone Composites: Autonomous Healing and Rate-Dependent Mechanics via Dynamic Boron Coordination Networks

Hongwen Zeng[1,2], Yan Peng[2], Tao Liu[2], Lijuan Zhao[1,]* Fengshun Zhang[2,]*

[1] College of Chemistry and Materials Science, Sichuan Normal University, Chengdu 610066, China; 20231201054@stu.sicnu.edu.cn (H.Z.)
[2] Institute of Chemical Materials, China Academy of Engineering Physics, Mianyang 621010, China; yanpengcaep@163.com (Y.P.); liutaocaep@163.com (T.L.)

* Correspondence: zfs8505@163.com (F.Z.); lijuan_zhao@sicnu.edu.cn (L.Z.)

Characterizations

Figure S1. presents the cyclic compression curves of materials with varying numbers of layers at the same strain level. Figure S2. to Figure S5. illustrate the calculation methods for the energy dissipation coefficients of materials with different numbers of layers. Figure S6. and Figure S7. show the compression curves of materials with different numbers of layers at various strain levels. Consistent with the results of the cyclic compression, the results of the compression test indicate that the material is relatively uniform. Moreover, as the number of layers increases, the compressive strength of the material gradually decreases. Figure 8 depicts the compression curves of materials with different numbers of layers at a strain level of 20%. Figure S9. displays the images of samples with different numbers of layers after the drop weight test experiment. Figure S10. presents the optical microscope images of samples with different numbers of layers after the compression stress relaxation experiment.

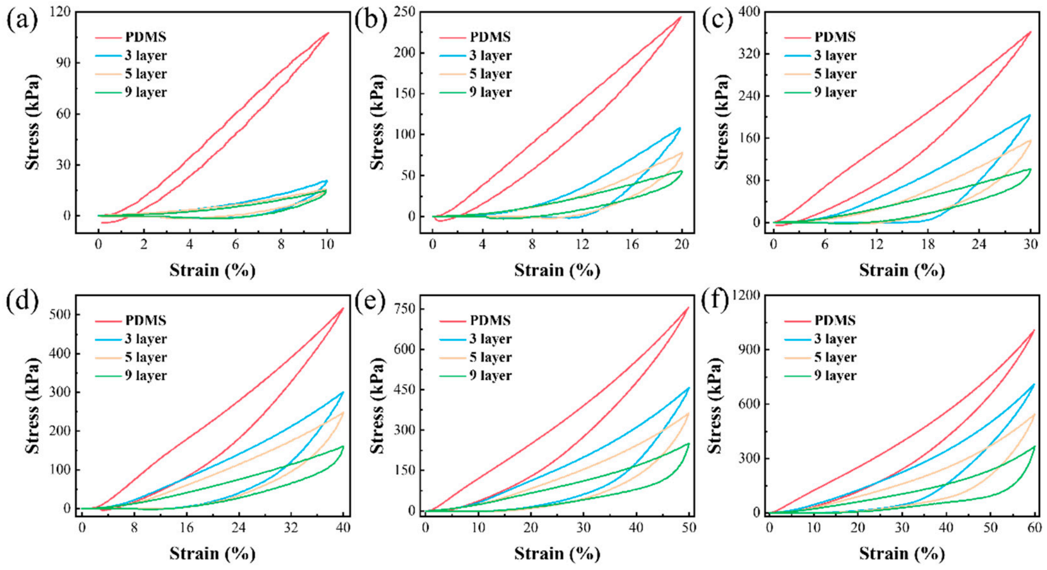

Figure S1. Compressive modulus of structures with different numbers of layers under various cyclic compression conditions: (a) 10%; (b) 20%; (c) 30%; (d) 40%; (e) 50%; (f) 60%.

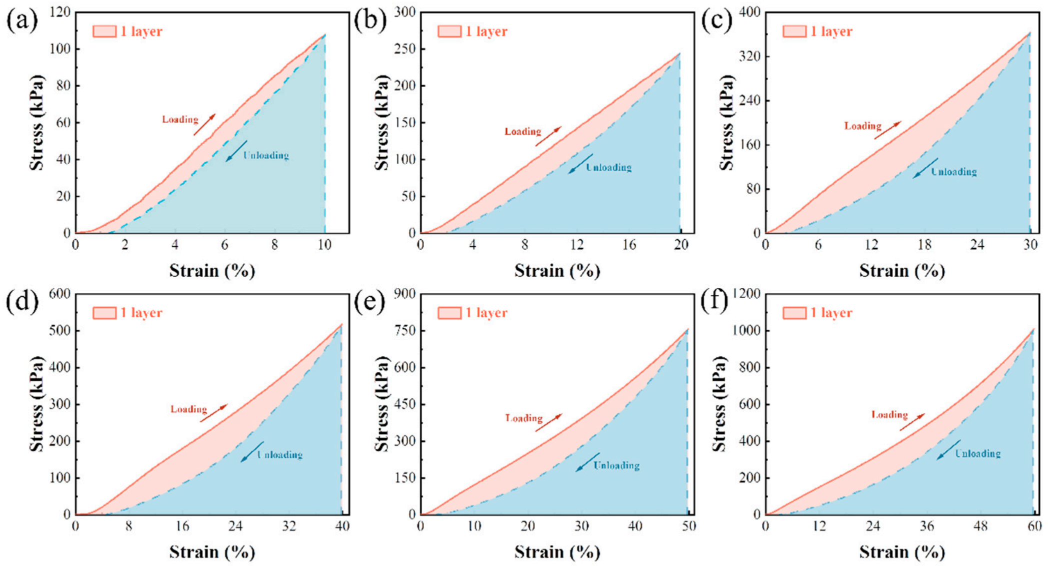

Figure S2. Energy dissipation in a PDMS structure under different cyclic compression conditions: (a) 10%; (b) 20%; (c) 30%; (d) 40%; (e) 50%; (f) 60%.

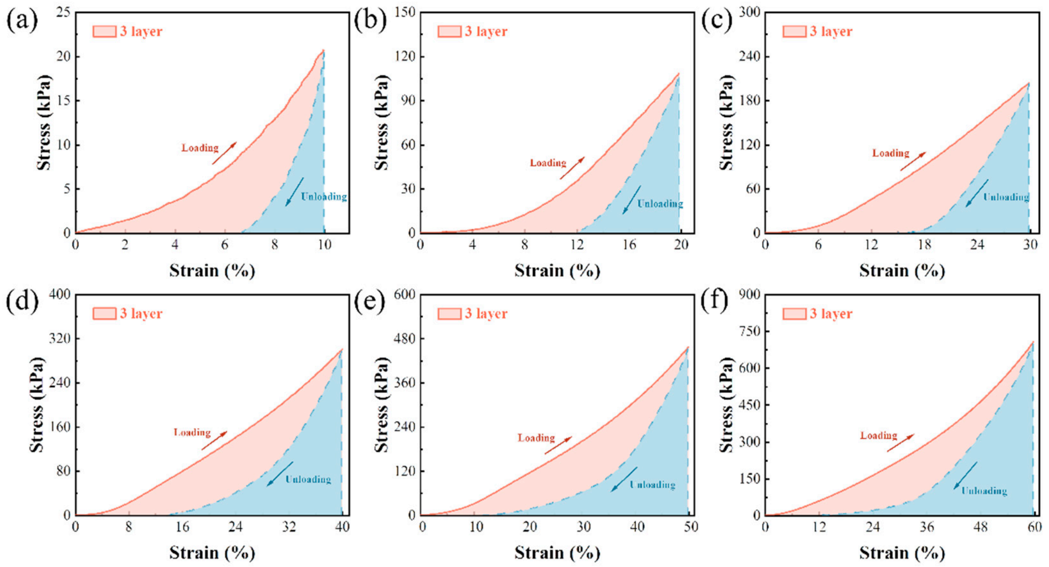

Figure S3. Energy dissipation in a 3-layer structure under different cyclic compression conditions: (a) 10%; (b) 20%; (c) 30%; (d) 40%; (e) 50%; (f) 60%.

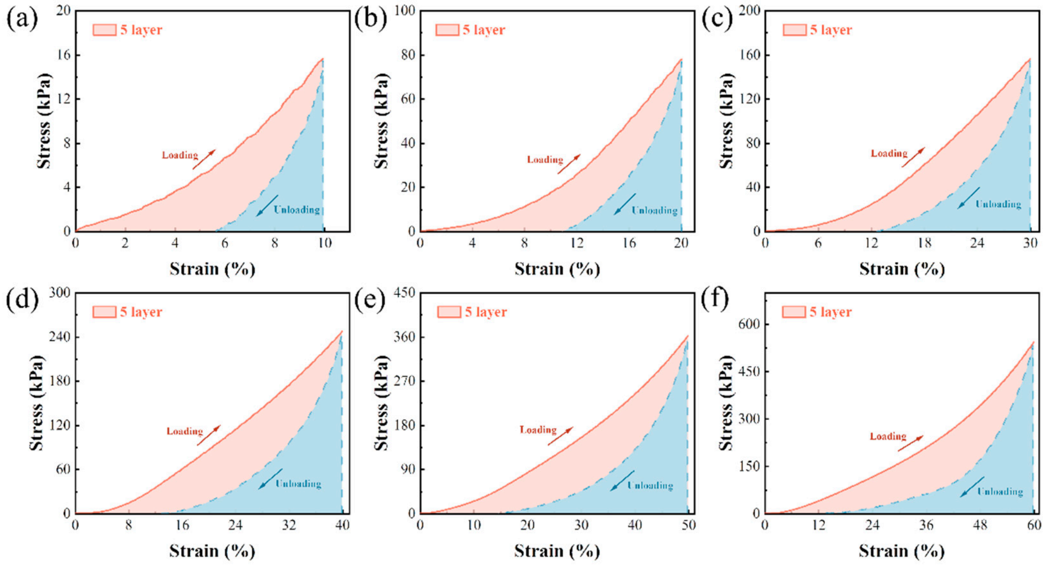

Figure S4. Energy dissipation in a 5-layer structure under different cyclic compression conditions: (a) 10%; (b) 20%; (c) 30%; (d) 40%; (e) 50%; (f) 60%.

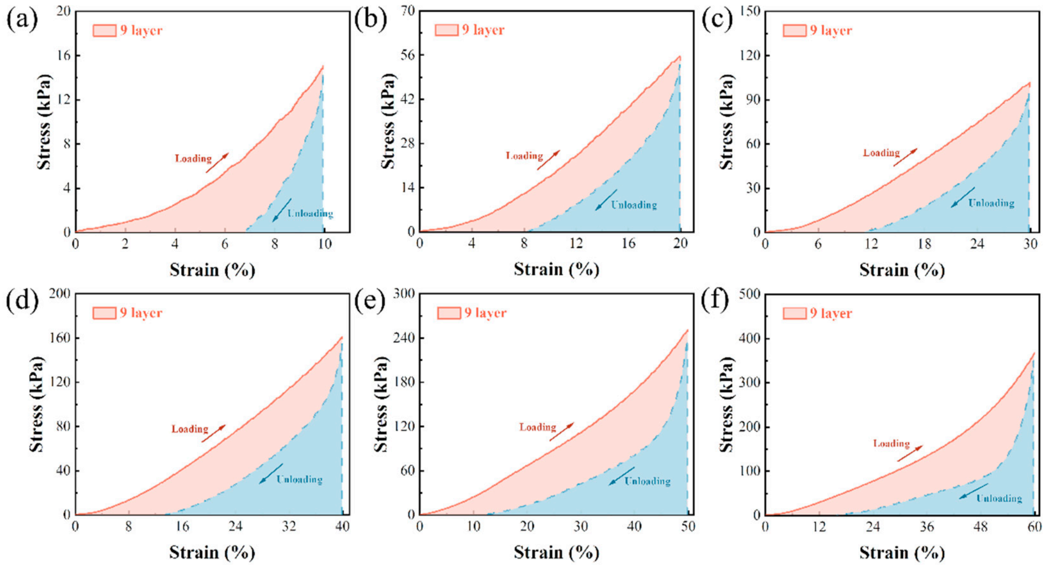

Figure S5. Energy dissipation in a 9-layer structure under different cyclic compression conditions: (a) 10%; (b) 20%; (c) 30%; (d) 40%; (e) 50%; (f) 60%.

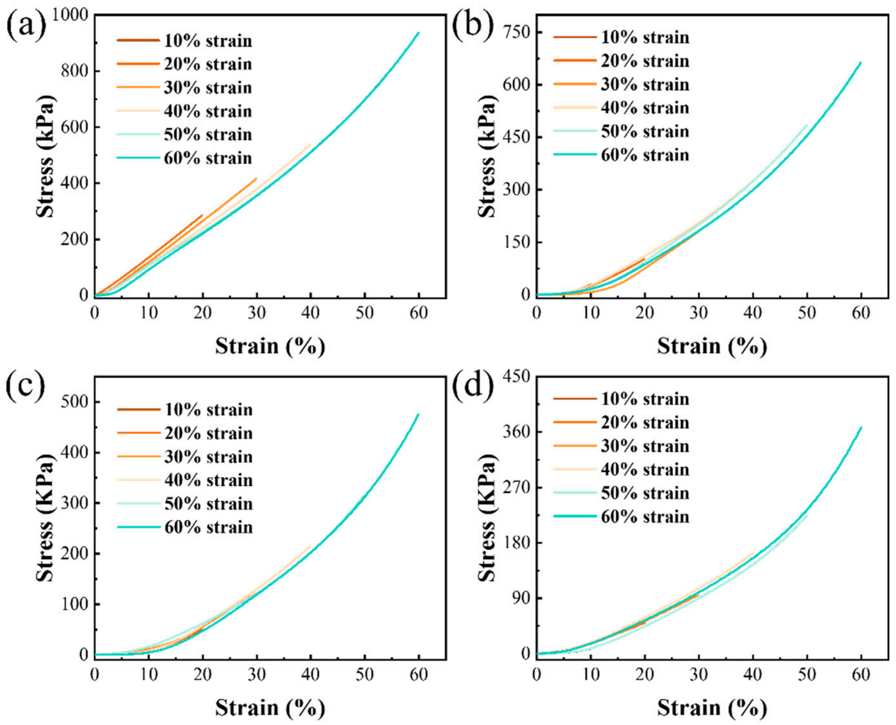

Figure S6. Compressive modulus of structures with different numbers of layers under various cyclic compression conditions: (a) PDMS; (b) 3-layer; (c) 5-layer; (d) 9-layer.

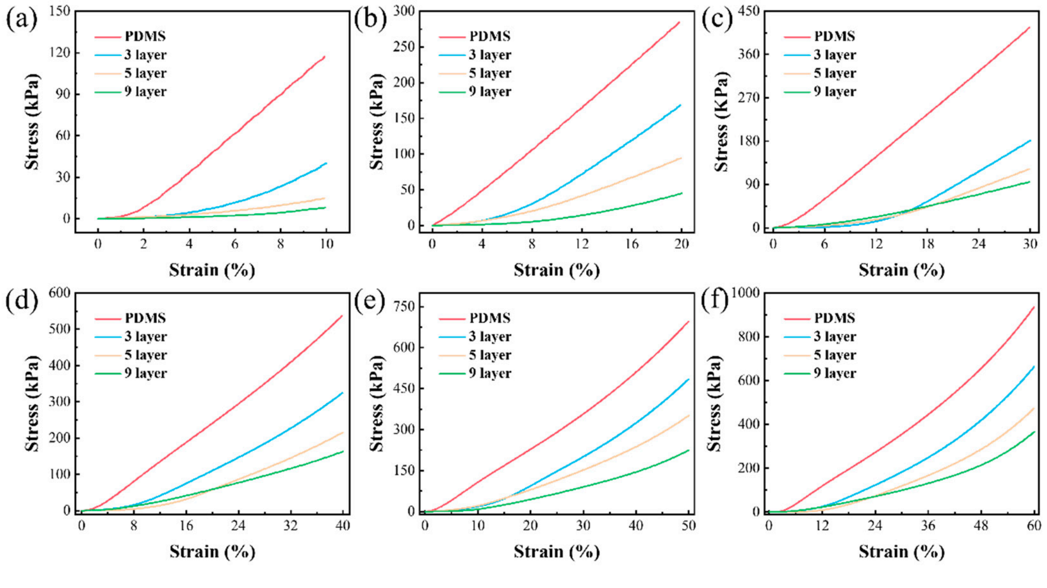

Figure S7. Compressive modulus of different multi-layer structures under varying compressive strain: (a) 10%; (b) 20%; (c) 30%; (d) 40%; (e) 50%; (f) 60%.

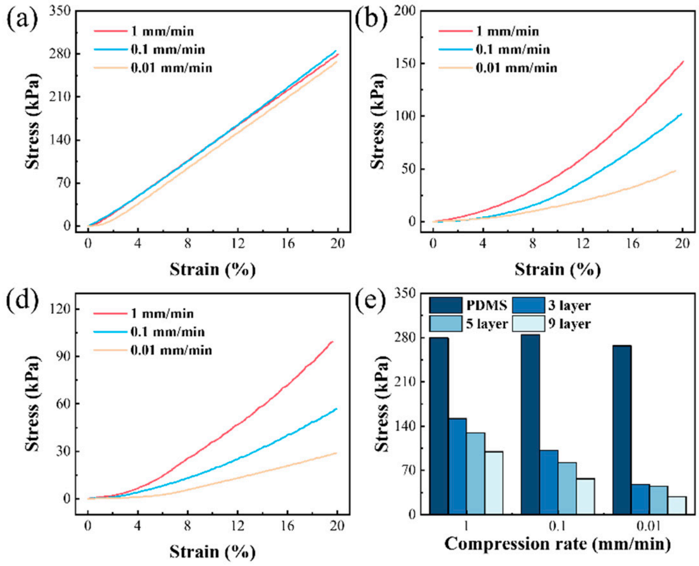

Figure S8. Compression behavior of multilayer structures at 20% strain under different strain rates: (a) PDMS; (b) 3-layer; (c) 5-layer; (d) 9-layer structures; (e) compression strength of different structures at various strain rates.

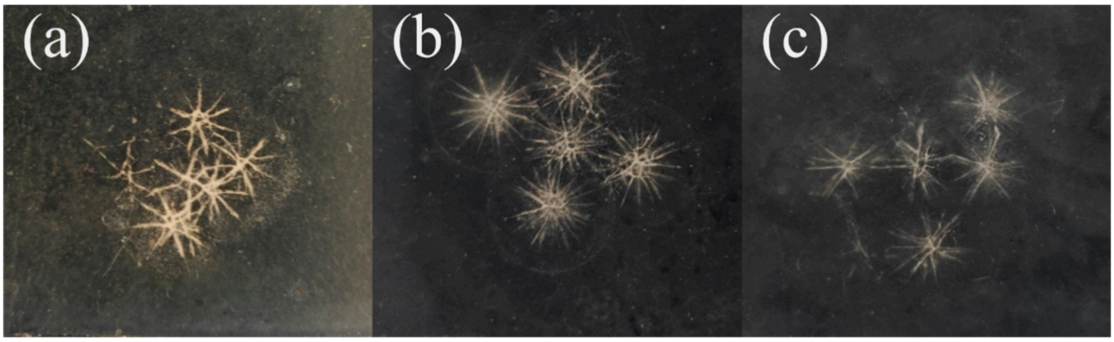

Figure S9. Images of impact-damaged specimens from drop-weight tests with different layer configurations: (a) 3-layer; (b) 5-layer; (c) 9-layer.

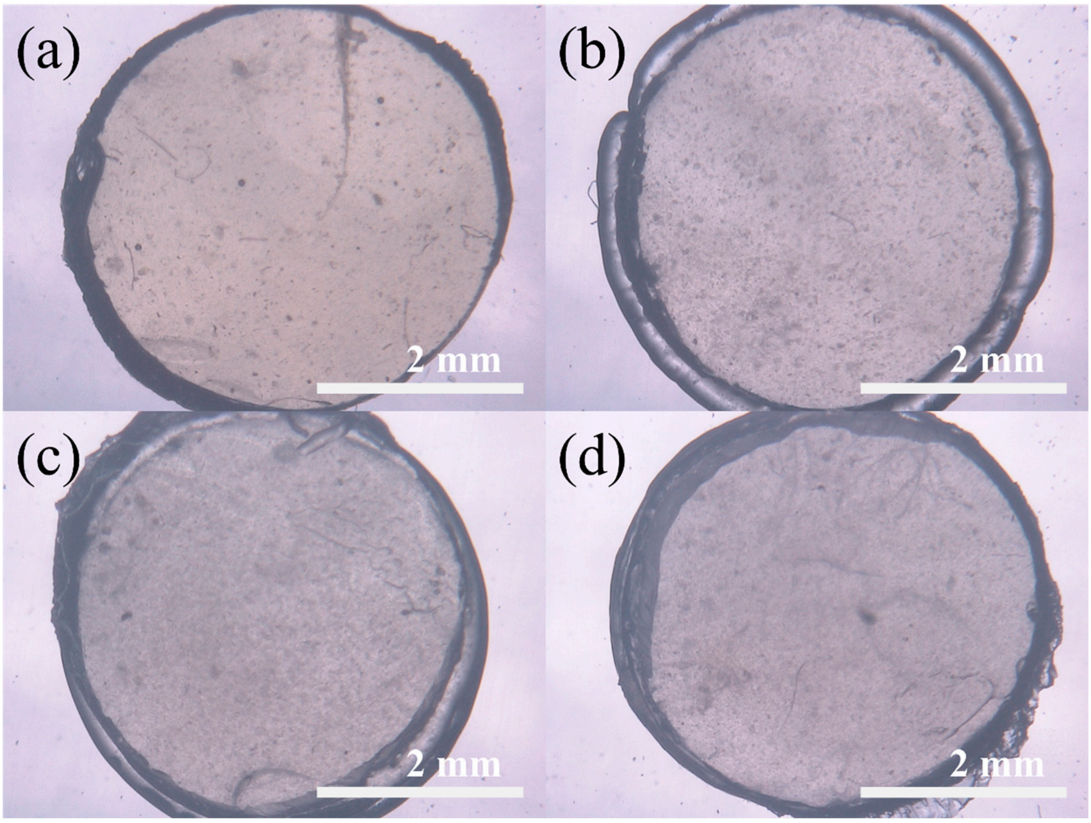

Figure S10. Optical microscopy images of compressive stress relaxation: (a) PDMS; (b) 3-layer; (c) 5-layer; (d) 9-layer.
